# Risk Factors of Central Lymph Node Metastasis in Papillary Thyroid Microcarcinoma and the Value of Sentinel Lymph Node Biopsy

**DOI:** 10.3389/fsurg.2021.680493

**Published:** 2021-06-16

**Authors:** Jing Zhao, Ye Zhao, Yuwei Ling, Hua Kang

**Affiliations:** Center for Thyroid and Breast Surgery, Department of General Surgery, Xuanwu Hospital, Capital Medical University, Beijing, China

**Keywords:** papillary thyroid microcarcinoma, risk factors, sentinel lymph node biopsy, positive sentinel lymph node ratio, additional positive lymph nodes

## Abstract

**Objective:** The present study aims to investigate the risk factors of central lymph node metastasis (CNM) in papillary thyroid microcarcinoma (PTMC) and evaluate the predictive value of sentinel lymph node biopsy (SLNB) during surgery.

**Methods:** The clinicopathological data of 179 patients with PTMC staging in cN0 and with SLNB performed were analyzed retrospectively. Positive sentinel lymph node ratio (PSLNR) and additional positive lymph node (APLN) were analyzed in cases with positive SLNB. The efficiency of SLNB was investigated. ROC curves were plotted to evaluate the predictive value of PSLNR for APLN.

**Results:** Cumulative maximum diameter of tumors (CMD) (*P* = 0.041) and capsule involvement (CI) (*P* = 0.014) were independent risk factors for central lymph node metastasis. The SLNB success rate was 97.28%, and the incidence of CNM was 31.28%. The sensitivity, specificity, false positive rate (FPR), false negative rate (FNR), positive predictive value (PPV), and negative predictive value (NPV) of SLNB to evaluate CNM and APLN were 82.14 vs. 61.54%, 100 vs. 80.39%, 0 vs. 19.61%, 17.86 vs. 38.46%, 100 vs. 34.78%, and 92.48 vs. 92.48%, respectively. For cases with positive SLNB, subgroup analysis was performed according to APLN. The PSLNRs of true and false positive groups were 0.4620 ± 0.1744 and 0.2425 ± 0.1355, respectively (*P* < 0.001). Analyzing the predictive value of PSLNR by the ROC curve, the optimal diagnostic cutoff point was 0.2917 [AUC = 0.861 (95% CI: 0.757, 0.966), *P* < 0.001], and the sensitivity, specificity, FPR, FNR, PPV, and NPV of PSLNR were 87.50, 73.33, 26.67, 12.50, 63.64, and 91.67%, respectively.

**Conclusion:** CMD and CI are independent risk factors for central lymph node metastasis in PTMC. SLNB has good predictive value for CNM. For cases with positive SLNB, PSLNR could be used to predict the presence of APLN, which may provide a theoretical basis for intraoperative lymph node dissection.

## Introduction

Thyroid cancer is one of the most common malignant tumors of the endocrine system and the incidence has doubled in recent years (1, 2). Thyroid cancer showed a rapid growth trend in Chinese women according to the data published in 2016 (3). In 1988, thyroid microcarcinoma was defined as thyroid cancer with a diameter not larger than 1.0 cm (4), which was accounted for about 35% of all thyroid cancers (5). Due to the application of high-resolution ultrasound and fine needle aspiration (FNA), the proportion of thyroid microcarcinoma increased gradually (6, 7). More than 50% of initially diagnosed cases of thyroid cancer are thyroid microcarcinoma (8). Thyroid microcarcinoma has a good prognosis, and the 5-year survival rate can reach 97% (9, 10). Surgery is still the most important treatment for thyroid carcinoma. However, how to assess the cervical central lymph node (CCLN) is still widely controversial. In breast cancer, SLNB has been widely accepted to evaluate the axillary lymph node status, but there was insufficient evidence to propagate the use of SLNB in papillary thyroid microcarcinoma. In our present study, we retrospectively analyzed the clinical data of PTMC patients in the Center for Thyroid and Breast Surgery of Xuanwu Hospital of Capital Medical University from 2013 to 2016 to investigate the risk factors of CCLN metastasis and evaluate the value of SLNB in predicting the risk of CCLN metastasis.

## Materials and Methods

### Patient Selection

A retrospective analysis was conducted on consecutive patients diagnosed with PTMC, who were admitted to the Center for Thyroid and Breast Surgery, Xuanwu Hospital, from August 2013 to December 2016. This study was conducted in accordance with the Declaration of Helsinki (as revised in 2013) and approved by the Ethical Review Board of Xuanwu Hospital of Capital Medical University (registration number: [2020]055). The clinicopathologic features were recorded and analyzed. The inclusion criteria were as follows: (1) papillary thyroid carcinoma was confirmed by FNAB before operation; (2) the maximum diameter of the tumor was ≤ 1.0 cm; (3) the newly diagnosed cases were without cervical lymph node puncture, radiofrequency ablation, or other neck operations before; and (4) the preoperative evaluation of lymph node was staging in clinically node-negative (cN0) according to the criteria put forward by Kowalski et al. (11): no enlarged cervical lymph nodes were palpated or the maximum diameter of the enlarged lymph nodes was <2.0 cm; the maximum diameter of the enlarged lymph nodes was <1.0 cm; or there was no central liquefaction due to necrosis, peripheral enhancement, disappearance of paranodal fat space, or similar changes even though the maximum diameter ranged from 1.0 to 2.0 cm by imaging examination. The exclusion criteria were as follows: (1) the other types of thyroid cancer or completely benign lesion were confirmed by postoperative paraffin section; (2) no SLN was found during the operation; and (3) the patient was unable to receive surgical treatment for various reasons.

### Surgical Procedure

The consent form for the operation and intraoperative frozen pathological examination was signed before the operation, and the informed consent form for the retrospective clinical study was signed during follow-up. All operations were performed by well-trained surgeons. After the entire thyroid was exposed, nanocarbon suspension (LUMMY, Chongqing, China, 0.5 mL/ampoule) was injected at multiple points under the thyroid capsule with a 21-G needle. Injection sites were distributed in the ipsilateral lobe of the tumor and isthmus evenly. Generally, 0.2–0.3 mL carbon nanoparticles were sufficient to stain the diseased lobe and isthmus, and residual agentia was applied to the contralateral lobe following the same procedure once total thyroidectomy was unavoidable. The subcapsular lymphatic vessels could be black stained after local compression for 1–2 min. The surgeons immediately looked for black-stained lymph nodes or lymph nodes directed by black-stained lymphatic vessels in the loose connective tissue of the larynx, trachea, and paratracheal side for SLN resection. According to the location of the lesions and the results of SLNB, the operative procedure was decided as follows: (1) unilateral lobectomy with ipsilateral CCLN dissection was performed in patients with unilateral lesions and negative SLNB; (2) total thyroidectomy with bilateral CCLN dissection was performed in patients with unilateral lesions and positive SLNB; and (3) total thyroidectomy with bilateral CCLN dissection was performed in all patients with bilateral lesions. Frozen pathological examination of the SLNB was reported by experienced pathologists. All the resection specimens were confirmed by postoperative routine pathological examination.

### Statistical Analysis

The continuous quantitative variables were described through means and standard deviations, and chi-square test was used to compare the categorical variables. The *t*-test or rank sum test was used to compare the continuous variables of the two groups. The risk factors of CCLN metastasis were analyzed by multivariate logistic regression. The optimal cutoff and AUC were calculated by ROC curve to illustrate the predictive value of PSLNR to predict the presence of APLN. Statistical analysis was carried out through SPSS23.0 and the *P*-value was set at 0.05.

## Results

### Patients' General Characteristics

**Figure 1:**
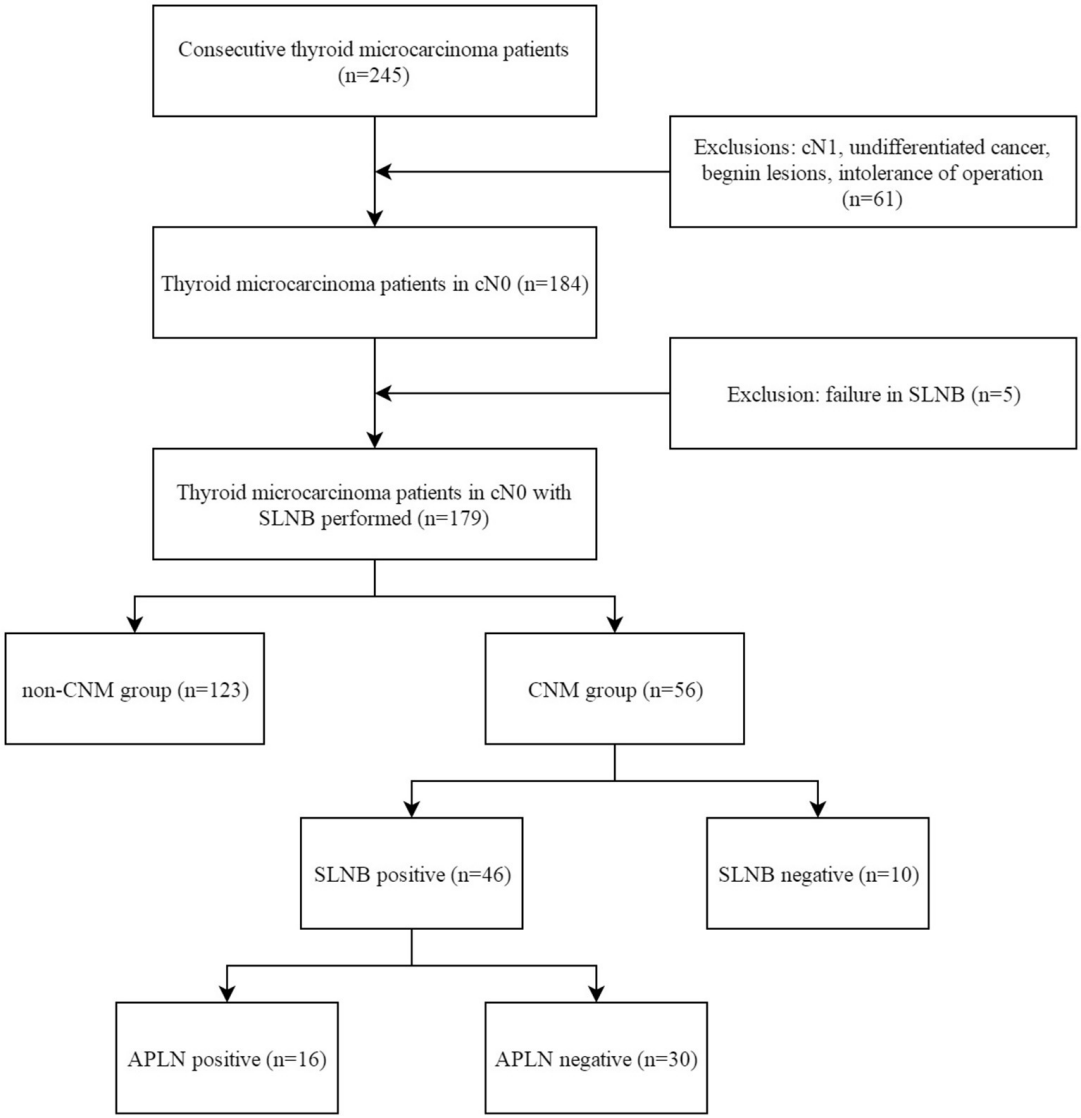
Flow diagram of the selection of patients. SLNB, sentinel lymph node biopsy; CNM, central lymph node metastasis; APLN, additional positive lymph node.

A total of 245 cases of PTMC admitted to the Center for Thyroid and Breast Surgery from August 2013 to December 2016 were retrospectively analyzed; 184 cases were staging in cN0 according to the clinical physical examination and imaging examination. SLNB failed in five cases, while SLNB was successfully incorporated into the group in 179 cases (Figure 1), and the average operation time was 148 (128, 176) min. There was no iatrogenic death and 25 cases had temporary hypoparathyroidism (13.97%) and seven cases had permanent hypoparathyroidism (3.91%). The success rate of biopsy was 97.28% (179/184). The status of the lymph nodes is shown in Table 1.

**Table 1:** Detection of lymph nodes in thyroid microcarcinoma.

**LN**		**Metastatic**	**Normal distribution**	**Median**	**Metastatic cases**	**Metastasis rate (%)**
		**LN**	**test**	**(upper and lower quartile)**		
SLN	897	69	*Z* = 1.933, *P* = 0.001	5 (3, 6)	46	25.70% (46/179)
CCLN	2,024	155	*Z* = 1.585, *P* = 0.013	10 (7, 14)	56	31.28% (56/179)

*LN, lymph node; SLN, sentinel lymph node; CCLN, cervical central lymph node*.

One hundred and seventy-nine consecutive patients who were diagnosed with PTMC (33 males and 146 females) were included in this study. The average age is 45.84 ± 11.71 years old. According to postoperative pathology, patients were divided into the CNM group (56 patients) and the non-CNM group (123 patients). The differences in age and sex were not statistically significant between the two groups, but significantly larger cumulative tumor diameter (*P* = 0.001) and higher CI proportion (*P* = 0.003) were present in the CNM group than in the non-CNM group (Table 2).

**Table 2:** Univariate analysis of CNM in papillary thyroid microcarcinoma.

**Risk factor**	**Metastasis**	**No metastasis**	**χ^**2**^ or *t*-test**	***P*-value**
Gender			1.238	0.266
Male	39.39% (13/33)	60.61% (20/33)		
Female	29.45% (43/146)	70.75% (103/146)		
Multifocality			3.128	0.077
Single	28.76% (44/153)	71.24% (109/153)		
Multiple	46.15% (12/26)	53.85% (14/26)		
Capsule involvement			8.643	0.003
Yes	39.45% (43/109)	60.55% (66/109)		
No	18.57% (13/70)	81.43% (57/70)		
Hashimoto thyroiditis			0.176	0.675
Yes	30.25% (36/119)	69.75% (83/119)		
No	33.33% (20/60)	66.67% (40/60)		
Location^a^			0.142	0.931
Upper pole	26.47% (9/34)	73.53% (25/34)		
Middle	29.13% (30/103)	70.87% (73/103)		
Lower pole	31.25% (5/16)	68.75% (11/16)		
Age	43.93 ± 12.60	46.72 ± 11.23	1.481	0.140
TSH level^b^ (μIU/mL)	1.87 (1.16, 2.47)	1.72 (1.24, 2.41)	0.132	0.716
Tumor (cumulative) maximum diameter^b^ (cm)	0.8 (0.7, 1.0)	0.7 (0.5, 0.9)	10.231	0.001

a *To explore the effect of location on CCLN metastasis only for a single lesion, the upper pole above the isthmus level, the lower pole below the isthmus level, and the middle between them were used as reference points*.

b *The measurement data with non-normal distribution were analyzed using the Kruskal–Wallis rank sum test*.

### Risk Factors of CNM

The risk factors were analyzed including age, sex, preoperative thyrotropin (TSH) level, CMD, single lesion location, and whether multifocal tumors, capsule involvement, and Hashimoto thyroiditis were present or not. Factors with statistical significance after screening by univariable regression analysis (*P*-value: entry 0.05, removal 0.10) were put into the multivariable regression model. The results showed that CMD (OR = 3.368, 95% CI: 1.049–10.809, *P* = 0.041) and CI (OR = 2.491, 95% CI: 1.200–5.169, *P* = 0.014) were independent factors predicting CCLN metastasis (Table 3).

**Table 3:** Multivariate analysis of CNM.

**Risk factors**	**Wald**	***P***	**OR**	**95% CI**	
				**Lower**	**Upper**
Capsule involvement	6.002	0.014	2.491	1.200	5.169
Maximum (cumulative) tumor diameter	4.166	0.041	3.368	1.049	10.809

*OR, odds ratio; CI, confidence interval*.

### The Value of SLNB in Predicting the CLN Status

**Figure 2:**
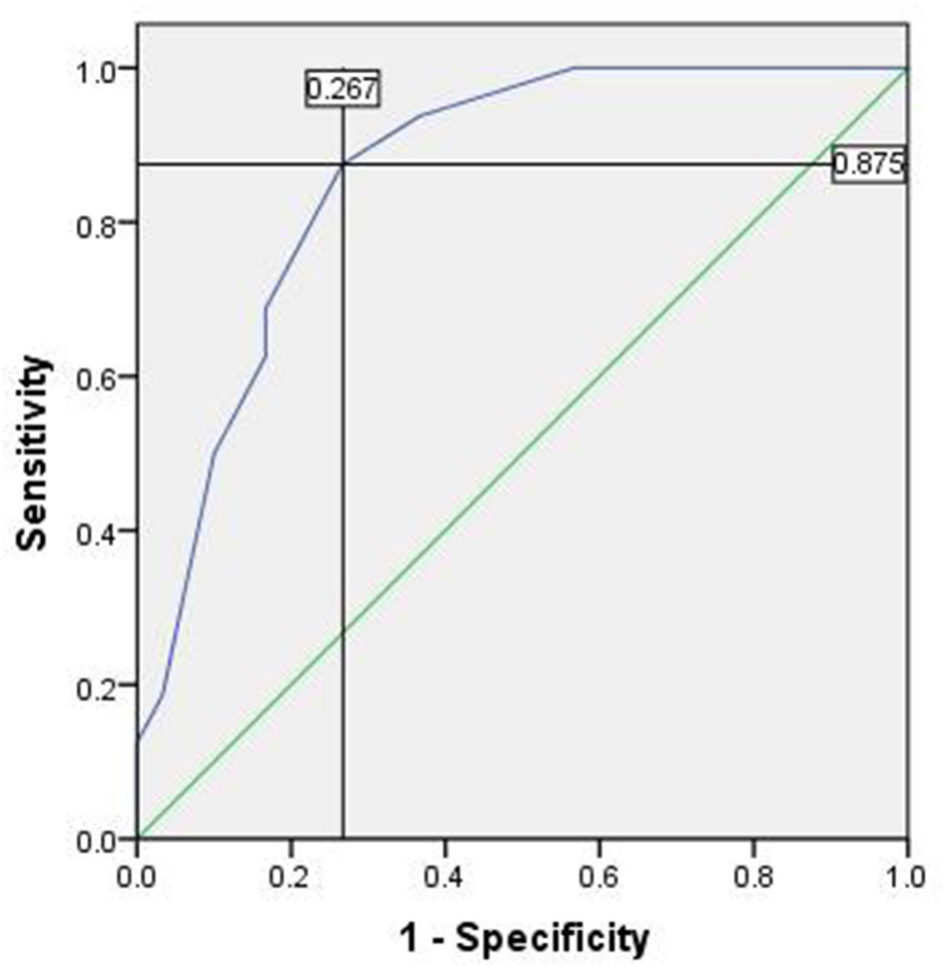
The ROC of PSLNR for predicting APLN. Area under the curve (AUC) was 0.861, *P* < 0.001, 95% CI (0.757, 0.966). The cutoff value was 0.2917 and the Youden index was 0.608. ROC, receiver operating characteristic curve; PSLNR, positive sentinel lymph node ratio; APLN, additional positive lymph node; AUC, area under the curve.

There were 46 cases with SLN metastasis and 56 cases with central metastasis confirmed by postoperative pathological examination, and 26 cases with APLN were detected postoperatively but omitted by the biopsy. In 10 of the 26 cases, SLNB did not find any positive lymph nodes at all; in the other 16 cases, SLNB found part of the metastatic lymph node. Therefore, the SLN metastasis rate was 25.70% (46/179), the CCLN metastasis rate was 31.28% (56/179), and the APLN metastasis rate was 14.53% (26/179) (Table 4). The prediction value of the CCLN and APLN metastasis based on the SLNB is shown in Table 5. We further analyzed the predictive value of PSLNR in APLN using ROC curve analysis as shown in Figure 2. The results illustrated that when the PSLNR reached 0.2917, there was a higher risk of APLN metastasis (AUC = 0.861, *P* < 0.001). According to the status of APLN, 16 of 46 cases with SLN metastasis were positive (group A) and the other 30 cases were negative (group B). *t*-test illustrated that the PSLNR of group A was significantly higher (*P* < 0.001) as shown in Table 6. The results obtained using 0.2917 as the threshold to evaluate the predictive value of PSLNR for APLN status are shown in Table 7.

**Table 4:** Status of SLN, CCLN, and APLN.

**SLN**	**CCLN**	**CCLN**	**APLN**	**APLN**
	**positive**	**negative**	**positive**	**positive**
	56	123	26	153
Positive	46	0	16	30
Negative	10	123	10	123

*SLN, sentinel lymph node; CCLN, cervical central lymph node; APLN, additional positive lymph node*.

**Table 5:** Predictive value of SLN for CCLN and APLN metastasis.

	**Sensitivity**	**Specificity**	**False positive**	**False negative**	**Positive predictive**	**Negative predictive**
		**rate**	**rate**	**value**	**value**
CCLN positive	82.14%	100%	0%	17.86%	100%	92.48%
APLN positive	61.54%	80.39%	19.61%	38.46%	34.78%	92.48%

*SLN, sentinel lymph node; CCLN, cervical central lymph node; APLN, additional positive lymph node*.

**Table 6:** PSLNR comparison between groups according to APLN.

**PSLNR**	**APLN positive**	**APLN negative**	***t*-value**	***P*-value**
**Cutoff = 0.2917**	**Group A**	**Group B**		
	16	30		
PSLNR+ (>0.2917)	14	8		
PSLNR– (<0.2917)	2	22		
Mean (*x* ±*s*)^a^	0.4620 ± 0.1744	0.2425 ± 0.1355	4.73	0.000

*APLN, additional positive lymph node; PSLNR, positive sentinel lymph node ratio*.

a *The statistic of the nonparametric test was Z = 1.207, P = 0.109 > 0.05, indicating that PSLNR has a normal distribution*.

**Table 7:** The predictive value of PSLNR for the status of APLN.

	**Sensitivity**	**Specificity**	**False positive**	**False negative**	**Positive predictive**	**Negative predictive**
			**rate**	**rate**	**value**	**value**
APLN+	87.50%	73.33%	26.67%	12.50%	63.64%	91.67%

*APLN, additional positive lymph node; PSLNR, positive sentinel lymph node ratio*.

## Discussion

Our study analyzed retrospective data of 179 patients with PTMC. The results revealed that central lymph node metastasis was significantly associated with larger tumor size (or cumulative maximum diameter) and capsule involvement instead of any other poor clinicopathologic characteristics. Sentinel lymph node biopsy was satisfactory in evaluating central lymph node status. Of the patients with positive SLNB, additional metastatic lymph node was absent in patients with lower PSLNR. This finding may have the potential to change the strategy of central lymph node dissection.

Prophylactic central cervical lymph node dissection (CND) in patients with thyroid cancer is still controversial. Although the ATA guideline (2015 edition) states that thyroidectomy without prophylactic central neck dissection (PCND) is appropriate for small (T1 or T2), noninvasive, cN0 PTC (12), other guidelines take some more cautious and conservative viewpoints. The British Thyroid Association Guidelines for the Management of Thyroid Cancer (third edition) states that in the strategy of lymph node management for papillary microcarcinoma, PCND should be considered in patients with tumors that are multifocal, pT3, and with extrathyroidal spread. In such cases, personalized decision-making is recommended (13). The Thyroid Cancer Committee of the Chinese Anti-Cancer Association worked out the Expert Consensus of Diagnosis and Treatment for Papillary Thyroid Microcarcinoma (2016 edition). The consensus states that PCND is recommended for PTMC under the condition of technical support (8).

Li et al. (14) conducted a meta-analysis in which 25 studies with comparison between thyroidectomy + PCND and thyroidectomy alone were eligible and included. For both PTC and PTMC, the overall recurrence in the thyroidectomy + PCND group was significantly lower than that in the thyroidectomy alone group. The central compartment recurrence was significantly higher in the thyroidectomy alone group than in the thyroidectomy + PCND group (OR = 3.41, 95% CI: 2.00, 5.80, *P* < 0.00001). A meta-analysis including 14 studies published in *Thyroid* (15) suggested that the incidence of occult lymph node metastasis ranged from 23.5 to 82.4% and those who undergo thyroidectomy + PCND have a 35% reduction in risk of locoregional recurrence than those who undergo thyroidectomy alone. Meanwhile, the researchers also pointed out the bias of retrospective studies on the above conclusions. Feng et al. (16) published their study results in the journal of *Frontiers in Endocrinology*. They observed that 40.3% of the overt PTMC patients had lymph node involvement, which was consistent with the high rate of CNM (43 to 64%) of patients undergoing PCND in previous studies; thus, routine PCND could be recommended. A 60-year observation including 900 cases of thyroid microcarcinoma conducted by the Mayo Clinic found that one-third of cases of microcarcinoma had CNM at the time of initial treatment (17). Early studies suggested that CNM was related to local recurrence and distant metastases. The risk of cervical lymph node recurrence and distant metastasis increased 6.2-fold (*P* = 0.01) and 11.2-fold (*P* = 0.003), respectively, when CNM was present at diagnosis (18, 19). Most supporters believe that CND removes potential micrometastases, thereby reducing recurrence, improving survival, avoiding possible complications caused by secondary CND, and maintaining better postoperative low thyroglobulin, and it was more conducive to disease staging and planning the ^131^I dosage (20–22).

However, opponents of CND demonstrate that unnecessary central lymph node dissection not only fails to benefit patients significantly but also increases the risk of surgical complications. Ito et al. investigated the clinical significance of lymph nodes in the central compartment of 600 patients and found that neither ultrasound-diagnosed nor pathologically confirmed CNM affected the disease-free survival rate of PTMC patients (28). Wada et al. proposed that CND can increase the incidence of temporary recurrent laryngeal nerve injury, hypocalcemia, and postoperative bleeding based on the investigation of 259 thyroid microcarcinoma patients (24). Lee et al. found that the incidence of temporary hypoparathyroidism in thyroidectomy with or without CND was 36.6 and 20.3%, respectively, in a prospective randomized controlled study (*P* = 0.043) (25). A retrospective study including 1,087 patients suggested that bilateral CND significantly increased the incidence of permanent hypoparathyroidism (16.2%), and even unilateral CND increased the incidence of temporary hypoparathyroidism (36.1%) (26). Although the incidence rate of hypothyroidism was lower in our study than in previous studies, the results illustrated the same trend. Therefore, it is fairly necessary to effectively determine the status of cervical central lymph nodes intraoperatively and avoid unnecessary lymph node dissection.

Although imaging examination, especially high-resolution ultrasound, is helpful to evaluate the preoperative cervical lymph node status of thyroid microcarcinoma, the accuracy is only 48.3%, while the sensitivity for predicting central lymph node metastasis is only 22.6–55% (27). The potential risk factors of central lymph node metastasis of thyroid microcarcinoma, such as age, sex, tumor size, capsule invasion, tumor location, multifocal tumors, inflammation, and TSH level, have been analyzed by several studies. Older patients have a lower rate of lymph node metastasis than younger patients (28–31). Additionally, lymph node metastasis is more likely to occur in male patients (32–36). However, the difference of CCLN metastasis between age and sex was not significant in our study. The location of thyroid microcarcinoma is related to CCLN metastasis (24). Tumors located in the inferior one-third of the lobe and isthmus are risk factors of CCLN metastasis (37, 38). However, there was no consensus about the division of thyroid lobes. In our present study, the thyroid lobe was divided into three parts referred to the isthmus of the gland, but our results showed that there was no significant correlation between CCLN metastasis and the tumor location. It is confirmed that tumor size, capsule involvement or extraglandular invasion, and multifocal tumors are risk factors of CCLN metastasis (39–48). Consistent with our present results, capsule involvement and tumor size were independent risk factors according to the multivariate regression analysis. Previous studies suggested that CNM was more likely to occur when the tumor size exceeded a threshold (31, 39–41, 44, 45). We used a ROC curve to determine the cutoff value of cumulative maximum diameter of tumors for CNM prediction. The curve reached the inflection point when the cutoff value was 0.75; however, the area under curve was only 0.648. In our opinion, further studies are needed to quantify this risk factor. Microcarcinoma is just a morphological concept and defined as a carcinoma of 10 mm size and below in the greatest dimension regardless of other histological features. Neuhold et al. (49) found that about 10.8% patients with PTMC had a superficial localization of the tumor, and superficial localization was significantly associated with positive lymph node (42.9 vs. 15.3%, *P* = 0.006). Li et al. (47) found that capsular invasion was not rare (13.7%) and an important prognostic characteristic of CNM, which was consistent with the rate reported by previous studies (9.9–26.8%). Vasileiadis et al. (50) studied 276 cases of PTMC and found that the rate of thyroid capsular invasion was 7.2% (20/276) and capsule invasion was an independent risk factor for CNM. Additionally, of the patients with tumor size larger than 5 mm, the rate of capsular invasion was 23% significantly higher than that of patients with tumor size of 5 mm and below (*P* = 0.001). A currently retrospective chart review of 182 PTMC patients found that minimal extrathyroidal extension (ETE) rate was as high as 37.9% (69/182) (51). Some other researchers have divided PTMCs into two separate groups. The first group contains PTMCs with lymph node metastasis and distant metastasis or any other malignant tumor symptoms and is called the symptomatic group. The group without any cancer symptoms, such as CNM or distant metastasis, is called the asymptomatic group (52). Sugitani et al. presented that 38% of symptomatic PTMC patients had recurrence and 8% died of primary disease. Almost 78% had poor prognostic factors such as ETE, extranodal invasion, metastatic lymph node size above 20 mm, and anaplastic components in the primary tumor. In our study, the rate of capsule involvement was 60.89% (109/179) higher than that in other previous studies. Considering the nature of retrospective study, there could be some selection bias unavoidably. It was certain that at the level of collecting data, all the participants in our study strived for objectivity and all the enrolled cases were consecutive. We sent a special researcher to the pathology department in order to verify the postoperative pathological result of each case. Objectively, 94 of the patients with capsule involvement had a maximum (cumulative) tumor diameter >5 mm, and the other 15 patients had smaller tumor size. We suspected that the high ratio of capsule involvement was a result of the combination of relatively larger tumor size (>5 mm) and probably more superficial localization.

The initial observation of sentinel lymph nodes (SLN) of parotid tumor was reported in 1960 (53), and in 1977, Cabanas (54) put forward the concept of SLN in the diagnosis and treatment of penile cancer. As a standard surgical procedure, SLNB has been widely applied in the treatment of breast cancer, melanoma, etc. As early as 1970, Noguchi et al. proposed that CCLN is the first echelon of cervical lymph node metastasis of thyroid cancer (55) and then Kelemen et al. firstly applied SLNB in thyroid malignancy treatment in 1998 (56). Wada et al. proposed that for thyroid microcarcinoma, CNM most likely involved pretracheal lymph nodes, followed by the ipsilateral central area, while contralateral CNM accounted for only 18.9%, and real jump metastasis was rare (24). Mercante et al. (57) also proposed that Delphian lymph nodes were the earliest lymph nodes invaded by primary cancer, and their biopsies could accurately and comprehensively reflect the regional lymphatic metastasis of thyroid cancer. We proposed a hypothesis that SLNB could be a satisfactory change in practice based on the importance and limitation of CND. Our results showed that the sensitivity, specificity, false positive rate, false negative rate, positive predictive value, and negative predictive value of evaluating CCLN status by SLNB were 82.14, 100, 0, 17.86, 100, and 92.48%, respectively. Similar to our present study, Fang et al. (58) reported the application of nanocarbon suspension in SLNB, the PPV of SLNB to predict the CNM was 86.7%, the NPV was 85.7%, and the FNR was 14.3%, which demonstrated that SLNB had an important predictive value for CNM in microcarcinoma and non-microcarcinoma. Simultaneously, SLNB is effective in predicting the CNM of papillary thyroid microcarcinoma, with a sensitivity of 90%, specificity of 100%, NPV of 98%, and PPV of 100% (59). Amir et al. reported that in all of 157 cases with SLNB conducted, there were only three cases of false negative, of which one case found the APLN while the positive SLNs were found in other two cases in postoperative paraffin pathology. The results suggested that pathologists are important for SLNB accuracy and also suggested that the process of SLNB has already treated most of the potential metastatic lymph nodes (59). It is worth mentioning that the false negative rate of APLN is as high as 38.46% if only predicting APLN by SLN metastasis. In recent years, the numbers of lymph node metastases (60) and the lymph node metastasis rate (MLNR) were used to describe lymph node status. A study including 573 cases of microcarcinoma found that the MLNR was an independent factor predicting local recurrence. Lang et al. reported that a higher MLNR might indicate residual or recurrent lesions (61). Another study illustrated that MLNR ≥0.42 was a predicting factor of tumor-related death (62). Inspired by the published literature, we further analyzed the predictive value of the PSLNR for APLN. In our study, 46 SLNB positive cases were divided into two groups in detail previously, and the PSLNR of group A was higher significantly (*P* < 0.001). ROC curve analysis showed an effective predictive value of PSLNR. When PSLNR reached 0.2917, APLN more likely occurred. Our results make a point of view that when the PSLNR is lower than 0.2917, metastatic lymph node residue is less likely to occur. In conclusion, for the management of lymph nodes in PTMC patients with lower PSLNR, unilateral CND even SLNB might be sufficient and safe.

Our study has some limitations: this was a single-center retrospective study; a multicenter study with a larger number is necessary to further confirm the value of SLNB in thyroid microcarcinoma. Among the 179 cases of PTMC included in our study, a longer follow-up period was necessary to discuss the relationship between SLNB and survival and recurrence.

In conclusion, tumor size and capsule involvement are independent risk factors for central lymph node metastasis in PTMC. Intraoperative SLNB can effectively predict the status of central cervical lymph nodes and reduce the risk of residual metastatic lymph nodes, as well as avoid unnecessary lymph node dissection in thyroid carcinoma patients.

## Data Availability Statement

The raw data supporting the conclusions of this article will be made available by the authors, without undue reservation.

## Ethics Statement

The studies involving human participants were reviewed and approved by Ethical Review Board of Xuanwu Hospital of Capital Medical University. Written informed consent for participation was not required for this study in accordance with the national legislation and the institutional requirements.

## Author Contributions

JZ and HK conceived and designed the study. YZ acquired all the raw data. JZ and YZ analyzed and interpreted the data. JZ wrote and reviewed the manuscript. YL revised the manuscript and performed English language editing. All authors read and approved the final version of the manuscript.

## Conflict of Interest

The authors declare that the research was conducted in the absence of any commercial or financial relationships that could be construed as a potential conflict of interest.

## References

[1] Londero SC Krogdahl A Bastholt L Overgaard J Trolle W Pedersen HB. Papillary thyroid microcarcinoma in Denmark 1996-2008: a national study of epidemiology and clinical significance. Thyroid. (2013) 23:1159–64. 10.1089/thy.2012.0595 23427917

[2] Chen AY Jemal A Ward EM. Increasing incidence of differentiated thyroid cancer in the United States, 1988-2005. Cancer. (2009) 115:3801–7. 10.1002/cncr.24416 19598221

[3] Chen W Zheng R Baade PD Zhang S Zeng H Bray F. Cancer statistics in China, 2015. CA Cancer J Clin. (2016) 66:115–32. 10.3322/caac.21338 26808342

[4] Hedinger C Williams ED Sobin LH. The WHO histological classification of thyroid tumors: a commentary on the second edition. Cancer. (1989) 63:908–11. 10.1002/1097-0142(19890301)63:5<908::aid-cncr2820630520>3.0.co;2-i 2914297

[5] Roti E degli Uberti EC Bondanelli M Braverman LE. Thyroid papillary microcarcinoma: a descriptive and meta-analysis study. Eur J Endocrinol. (2008) 159:659–73. 10.1530/EJE-07-0896 18713843

[6] Xiang Y Lin K Dong S Qiao LI He Q Zhang X. Prediction of central lymph node metastasis in 392 patients with cervical lymph node-negative papillary thyroid carcinoma in Eastern China. Oncol Lett. (2015) 10:2559–64. 10.3892/ol.2015.3544 26622889 PMC4580067

[7] Cheng P Chen ED Zheng HM He QX Li Q. Ultrasound score to select subcentimeter-sized thyroid nodules requiring ultrasound-guided fine needle aspiration biopsy in eastern China. Asian Pac J Cancer Prev. (2013) 14:4689–92. 10.7314/APJCP.2013.14.8.4689 24083727

[8] Gao M Ge MH Ji QH Xu ZG Lu HK Cheng RC Guan HX. Chinese expert consensus on the diagnosis and treatment of thyroid micropapillary carcinoma (version 2016). Clin Oncol China. (2016) 43:405–11. 10.20892/j.issn.2095-3941.2017.0051

[9] Pazaitou-Panayiotou K Capezzone M Pacini F. Clinical features and therapeutic implication of papillary thyroid microcarcinoma. Thyroid. (2007) 17:1085–92. 10.1089/thy.2007.0005 18047430

[10] Hakala T Kellokumpu-Lehtinen P Kholová I Holli K Huhtala H Sand J. Rising incidence of small size papillary thyroid cancers with no change in disease-specific survival in Finnish thyroid cancer patients. Scand J Surg. (2012) 101:301–6. 10.1177/145749691210100415 23238509

[11] Kowalski LP Bagietto R Lara JR Santos RL Silva JF Jr., Magrin J. Prognostic significance of the distribution of neck node metastasis from oral carcinoma. Head Neck. (2000) 22:207–14. 10.1002/(sici)1097-0347(200005)22:3<207::aid-hed1>3.0.co;2-9 10748442

[12] Haugen BR Alexander EK Bible KC Doherty GM Mandel SJ Nikiforov YE. 2015 American thyroid association management guidelines for adult patients with thyroid nodules and differentiated thyroid cancer: the American thyroid association guidelines task force on thyroid nodules and differentiated thyroid cancer. Thyroid. (2016) 26:1–133. 10.1089/thy.2015.0020 26462967 PMC4739132

[13] Perros P Boelaert K Colley S Evans C Evans RM Gerrard Ba G. Guidelines for the management of thyroid cancer. Clin Endocrinol (Oxf). (2014) 81(Suppl. 1):1–122. 10.1111/cen.12515 24989897

[14] Liu H Li Y Mao Y. Local lymph node recurrence after central neck dissection in papillary thyroid cancers: a meta analysis. Eur Ann Otorhinolaryngol Head Neck Dis. (2019) 136:481–7. 10.1016/j.anorl.2018.07.010 31196800

[15] Lang BH Ng SH Lau LL Cowling BJ Wong KP Wan KY. A systematic review and meta-analysis of prophylactic central neck dissection on short-term locoregional recurrence in papillary thyroid carcinoma after total thyroidectomy. Thyroid. (2013) 23:1087–98. 10.1089/thy.2012.0608 23402640

[16] Feng JW Pan H Wang L Ye J Jiang Y Qu Z. Determine the optimal extent of thyroidectomy and lymphadenectomy for patients with papillary thyroid microcarcinoma. Front Endocrinol (Lausanne). (2019) 10:363. 10.3389/fendo.2019.00363 31275239 PMC6593058

[17] Hay ID Hutchinson ME Gonzalez-Losada T McIver B Reinalda ME Grant CS. Papillary thyroid microcarcinoma: a study of 900 cases observed in a 60-year period. Surgery (Oxf). (2008) 144:980–7. 10.1016/j.surg.2008.08.035 19041007

[18] Chow SM Law SC Chan JK Au SK Yau S Lau WH. Papillary microcarcinoma of the thyroid-Prognostic significance of lymph node metastasis and multifocality. Cancer. (2003) 98:31–40. 10.1002/cncr.11442 12833452

[19] Simon D Goretzki PE Witte J Röher HD. Incidence of regional recurrence guiding radicality in differentiated thyroid carcinoma. World J Surg. (1996) 20:860–6. 10.1007/s002689900131 8678963

[20] Gyorki DE Untch B Tuttle RM Shaha AR. Prophylactic central neck dissection in differentiated thyroid cancer: an assessment of the evidence. Ann Surg Oncol. (2013) 20:2285–9. 10.1245/s10434-013-2897-6 23417435

[21] Wang Q Chu B Zhu J Zhang S Liu Y Zhuang M. Clinical analysis of prophylactic central neck dissection for papillary thyroid carcinoma. Clin Transl Oncol. (2014) 16:44–8. 10.1007/s12094-013-1038-9 23606353 PMC3884135

[22] Bonnet S Hartl D Leboulleux S Baudin E Lumbroso JD Al Ghuzlan A. Prophylactic lymph node dissection for papillary thyroid cancer less than 2 cm: implications for radioiodine treatment. J Clin Endocrinol Metab. (2009) 94:1162–7. 10.1210/jc.2008-1931 19116234

[23] Baumgarten HD Bauer AJ Isaza A Mostoufi-Moab S Kazahaya K Adzick NS. Surgical management of pediatric thyroid disease: complication rates after thyroidectomy at the Children's Hospital of Philadelphia high-volume Pediatric Thyroid Center. J Pediatr Surg. (2019) 54:1969–75. 10.1016/j.jpedsurg.2019.02.009 30902456

[24] Wada N Duh QY Sugino K Iwasaki H Kameyama K Mimura T. Lymph node metastasis from 259 papillary thyroid microcarcinomas: frequency, pattern of occurrence and recurrence, and optimal strategy for neck dissection. Ann Surg. (2003) 237:399–407. 10.1097/01.SLA.0000055273.58908.19 12616125 PMC1514312

[25] Lee DY Oh KH Cho JG Kwon SY Woo JS Baek SK. The benefits and risks of prophylactic central neck dissection for papillary thyroid carcinoma: prospective cohort study. Int J Endocrinol. (2015) 2015:571480. 10.1155/2015/571480 26246805 PMC4515503

[26] Giordano D Valcavi R Thompson GB Pedroni C Renna L Gradoni P. Complications of central neck dissection in patients with papillary thyroid carcinoma: results of a study on 1087 patients and review of the literature. Thyroid. (2012) 22:911–7. 10.1089/thy.2012.0011 22827494

[27] Xue S Wang P Hurst ZA Chang YS Chen G. Active surveillance for papillary thyroid microcarcinoma: challenges and prospects. Front Endocrinol (Lausanne). (2018) 9:736. 10.3389/fendo.2018.00736 30619082 PMC6302022

[28] Ito Y Miyauchi A Kihara M Higashiyama T Kobayashi K Miya A. Patient age is significantly related to the progression of papillary microcarcinoma of the thyroid under observation. Thyroid. (2014) 24:27–34. 10.1089/thy.2013.0367 24001104 PMC3887422

[29] Cho JK Kim JY Jeong CY Jung EJ Park ST Jeong SH. Clinical features and prognostic factors in papillary thyroid microcarcinoma depends on age. J Korean Surg Soc. (2012) 82:281–7. 10.4174/jkss.2012.82.5.281 22563534 PMC3341476

[30] Lombardi CP Bellantone R De Crea C Paladino NC Fadda G Salvatori M. Papillary thyroid microcarcinoma: extrathyroidal extension, lymph node metastases, and risk factors for recurrence in a high prevalence of goiter area. World J Surg. (2010) 34:1214–21. 10.1007/s00268-009-0375-x 20052467

[31] Xu Y Xu L Wang J. Clinical predictors of lymph node metastasis and survival rate in papillary thyroid microcarcinoma: analysis of 3607 patients at a single institution. J Surg Res. (2018) 221:128–34. 10.1016/j.jss.2017.08.007 29229118

[32] Zhang Q Wang Z Meng X Duh QY Chen G. Predictors for central lymph node metastases in CN0 papillary thyroid microcarcinoma (mPTC): a retrospective analysis of 1304 cases. Asian J Surg. (2019) 42:571–6. 10.1016/j.asjsur.2018.08.013 30348606

[33] Cheng F Chen Y Zhu L Zhou B Xu Y Chen Y. Risk Factors for cervical lymph node metastasis of papillary thyroid microcarcinoma: a single-center retrospective study. Int J Endocrinol. (2019) 2019:8579828. 10.1155/2019/8579828 30774660 PMC6350584

[34] Sun W Lan X Zhang H Dong W Wang Z He L. Risk factors for central lymph node metastasis in CN0 papillary thyroid carcinoma: a systematic review and meta-analysis. PLoS ONE. (2015) 10:e0139021. 10.1371/journal.pone.0139021 26431346 PMC4592212

[35] Zhang LY Liu ZW Liu YW Gao WS Zheng CJ. Risk factors for nodal metastasis in cN0 papillary thyroid microcarcinoma. Asian Pac J Cancer Prev. (2015) 16:3361–3. 10.7314/APJCP.2015.16.8.3361 25921145

[36] Yang Y Chen C Chen Z Jiang J Chen Y Jin L. Prediction of central compartment lymph node metastasis in papillary thyroid microcarcinoma. Clin Endocrinol (Oxf). (2014) 81:282–8. 10.1111/cen.12417 24483297

[37] Xu D Lv X Wang S Dai W. Risk factors for predicting central lymph node metastasis in papillary thyroid microcarcinoma. Int J Clin Exp Pathol. (2014) 7:6199–205. 25337270 PMC4203241

[38] Cai YF Wang QX Ni CJ Zhang XJ Chen ED Dong SY. A scoring system is an effective tool for predicting central lymph node metastasis in papillary thyroid microcarcinoma: a case-control study. World J Surg Oncol. (2016) 14:45. 10.1186/s12957-016-0808-6 26911241 PMC4765135

[39] Liu Z Wang L Yi P Wang CY Huang T. Risk factors for central lymph node metastasis of patients with papillary thyroid microcarcinoma: a meta-analysis. Int J Clin Exp Pathol. (2014) 7:932–7. 24696711 PMC3971295

[40] Zhao Q Ming J Liu C Shi L Xu X Nie X. Multifocality and total tumor diameter predict central neck lymph node metastases in papillary thyroid microcarcinoma. Ann Surg Oncol. (2013) 20:746–52. 10.1245/s10434-012-2654-2 22972508

[41] Kim KE Kim EK Yoon JH Han KH Moon HJ Kwak JY. Preoperative prediction of central lymph node metastasis in thyroid papillary microcarcinoma using clinicopathologic and sonographic features. World J Surg. (2013) 37:385–91. 10.1007/s00268-012-1826-3 23073506

[42] Kim E Choi JY Koo do H Lee KE Youn YK. Differences in the characteristics of papillary thyroid microcarcinoma ≤ 5 mm and >5 mm in diameter. Head Neck. (2015) 37:694–7. 10.1002/hed.23654 24596325

[43] Haymart MR Cayo M Chen H. Papillary thyroid microcarcinomas: big decisions for a small tumor. Ann Surg Oncol. (2009) 16:3132–9. 10.1245/s10434-009-0647-6 19653044 PMC4852736

[44] Zheng X Peng C Gao M Zhi J Hou X Zhao J. Risk factors for cervical lymph node metastasis in papillary thyroid microcarcinoma: a study of 1,587 patients. Cancer Biol Med. (2019) 16:121–30. 10.20892/j.issn.2095-3941.2018.0125 31119052 PMC6528461

[45] Wang J Sun Y Shi L Xie L. Predictive factors for non-small-volume central lymph node metastases (more than 5 or ≥ 2 mm) in clinically node-negative papillary thyroid carcinoma. Medicine. (2019) 98:e14028. 10.1097/MD.0000000000014028 30608456 PMC6344183

[46] Jeon MJ Kim YN Sung TY Hong SJ Cho YY Kim TY. Practical initial risk stratification based on lymph node metastases in pediatric and adolescent differentiated thyroid cancer. Thyroid. (2018) 28:193–200. 10.1089/thy.2017.0214 29179646

[47] Li M Zhu XY Lv J Lu K Shen MP Xu ZL. Risk factors for predicting central lymph node metastasis in papillary thyroid microcarcinoma (CN0): a study of 273 resections. Eur Rev Med Pharmacol Sci. (2017) 21:3801–7. 28975988

[48] Mazeh H Samet Y Hochstein D Mizrahi I Ariel I Eid A. Multifocality in well-differentiated thyroid carcinomas calls for total thyroidectomy. Am J Surg. (2011) 201:770–5. 10.1016/j.amjsurg.2010.03.004 20864083

[49] Neuhold N Schultheis A Hermann M Krotla G Koperek O Birner P. Incidental papillary microcarcinoma of the thyroid–further evidence of a very low malignant potential: a retrospective clinicopathological study with up to 30 years of follow-up. Ann Surg Oncol. (2011) 18:3430–6. 10.1245/s10434-011-1663-x 21431405

[50] Vasileiadis I Karakostas E Charitoudis G Stavrianaki A Kapetanakis S Kouraklis G. Papillary thyroid microcarcinoma: clinicopathological characteristics and implications for treatment in 276 patients. Eur J Clin Invest. (2012) 42:657–64. 10.1111/j.1365-2362.2011.02633.x 22168782

[51] Kaliszewski K Diakowska D Rzeszutko M Nowak Ł Aporowicz M Wojtczak B. Risk factors of papillary thyroid microcarcinoma that predispose patients to local recurrence. PLoS ONE. (2020) 15:e0244930. 10.1371/journal.pone.0244930 33382852 PMC7775061

[52] Sugitani I Fujimoto Y. Symptomatic versus asymptomatic papillary thyroid microcarcinoma: a retrospective analysis of surgical outcome and prognostic factors. Endocr J. (1999) 46:209–16. 10.1507/endocrj.46.209 10426589

[53] Gould EA Winship T Philbin PH Kerr HH. Observations on a “sentinel node” in cancer of the parotid. Cancer. (1960) 13:77–8. 10.1002/1097-0142(196001/02)13:1<77::aid-cncr2820130114>3.0.co;2-d 13828575

[54] Cabanas RM. An approach for the treatment of penile carcinoma. Cancer. (1977) 39:456–66. 10.1002/1097-0142(197702)39:2<456::aid-cncr2820390214>3.0.co;2-i 837331

[55] Noguchi S Noguchi A Murakami N. Papillary carcinoma of the thyroid. I. Developing pattern of metastasis. Cancer. (1970) 26:1053–60. 10.1002/1097-0142(197011)26:5<1053::aid-cncr2820260513>3.0.co;2-x 5476786

[56] Kelemen PR Van Herle AJ Giuliano AE. Sentinel lymphadenectomy in thyroid malignant neoplasms. Arch Surg. (1998) 133:288–92. 10.1001/archsurg.133.3.288 9517742

[57] Mercante G Frasoldati A Pedroni C Formisano D Renna L Piana S. Prognostic factors affecting neck lymph node recurrence and distant metastasis in papillary microcarcinoma of the thyroid: results of a study in 445 patients. Thyroid. (2009) 19:707–16. 10.1089/thy.2008.0270 19348581

[58] Fang JG Luan XY Shen HM Wang C Tian AJ Cai SP. Clinical and pathological study of sentinel lymph nodes in thyroid carcinoma. J Cancer Prevent Treat. (2001) 8:149–50.

[59] Amir A Payne R Richardson K Hier M Mlynarek A Caglar D. Sentinel lymph node biopsy in thyroid cancer: it can work but there are pitfalls. Otolaryngol Head Neck Surg. (2011) 145:723–6. 10.1177/0194599811415809 21753032

[60] Lee YS Lim YS Lee JC Wang SG Kim IJ Lee BJ. Clinical implication of the number of central lymph node metastasis in papillary thyroid carcinoma: preliminary report. World J Surg. (2010) 34:2558–63. 10.1007/s00268-010-0749-0 20703463

[61] Lang BH Tang AH Wong KP Shek TW Wan KY Lo C. Significance of size of lymph node metastasis on postsurgical stimulated thyroglobulin levels after prophylactic unilateral central neck dissection in papillary thyroid carcinoma. Ann Surg Oncol. (2012) 19:3472–8. 10.1245/s10434-012-2385-4 22565664 PMC3442170

[62] Schneider DF Chen H Sippel RS. Impact of lymph node ratio on survival in papillary thyroid cancer. Ann Surg Oncol. (2013) 20:1906–11. 10.1245/s10434-012-2802-8 23263904 PMC3609925

